# Vitamin B_12_ release through bacteriophage-mediated cell lysis of the marine bacterium *Sulfitobacter* sp. M39

**DOI:** 10.1093/ismeco/ycaf136

**Published:** 2025-08-29

**Authors:** Sabiha Sultana, Stefan Bruns, Armando Pacheco-Valenciana, Maliheh Mehrshad, Heinz Wilkes, Meinhard Simon, Sarahi Garcia, Gerrit Wienhausen

**Affiliations:** Institute for Chemistry and Biology of the Marine Environment (ICBM), School of Mathematics and Science, Carl von Ossietzky Universität Oldenburg, Oldenburg, Germany; Institute for Chemistry and Biology of the Marine Environment (ICBM), School of Mathematics and Science, Carl von Ossietzky Universität Oldenburg, Oldenburg, Germany; Department of Ecology, Environment, and Plant Sciences, Science for Life Laboratory, Stockholm University, Stockholm, Sweden; Department of Aquatic Sciences and Assessment, Science for Life Laboratory, Swedish University of Agricultural Sciences, Uppsala, Sweden; Institute for Chemistry and Biology of the Marine Environment (ICBM), School of Mathematics and Science, Carl von Ossietzky Universität Oldenburg, Oldenburg, Germany; Institute for Chemistry and Biology of the Marine Environment (ICBM), School of Mathematics and Science, Carl von Ossietzky Universität Oldenburg, Oldenburg, Germany; Helmholtz Institute for Functional Marine Biodiversity at the University of Oldenburg (HIFMB), Oldenburg, Germany; Institute for Chemistry and Biology of the Marine Environment (ICBM), School of Mathematics and Science, Carl von Ossietzky Universität Oldenburg, Oldenburg, Germany; Department of Ecology, Environment, and Plant Sciences, Science for Life Laboratory, Stockholm University, Stockholm, Sweden; Helmholtz Institute for Functional Marine Biodiversity at the University of Oldenburg (HIFMB), Oldenburg, Germany; Institute for Chemistry and Biology of the Marine Environment (ICBM), School of Mathematics and Science, Carl von Ossietzky Universität Oldenburg, Oldenburg, Germany

**Keywords:** vitamin B_12_, cobalamin, bacteriophages, bacteriophage-mediated cell lysis, diatom, metabolite cross-feeding, marine microbial community, microbial interaction

## Abstract

Vitamin B_12_ (B_12_) is an essential cofactor for vital metabolic processes in both prokaryotes and eukaryotes. *De novo* B_12_ biosynthesis is exclusively carried out by a modicum of prokaryotes, although being required by most organisms. Recently, it has been demonstrated that not all B_12_-prototrophic bacteria voluntarily share this vital cofactor and, therefore, are termed B_12_-retainers. Consequently, low biosynthesis potential and limited voluntary release lead to a large discrepancy between availability and demand for B_12_ in the ocean, indicating that release of B_12_ may be an important control. Hence, in this study, we examined a specific release process, cell lysis after phage infection. We isolated bacteriophages specific for the B_12_-prototrophic, yet B_12_-retainer bacterium *Sulfitobacter* sp. M39. The addition of the bacteriophages to a *Sulfitobacter* sp. M39 mono-culture led to a significant increase in virus-like particles, reduced bacterial growth, and quantifiable extracellular dissolved B_12_. When introducing bacteriophages to a co-culture comprising the host bacterium and the B_12_-auxotrophic diatom *Thalassiosira pseudonana*, we observed rapid response in the form of microalgal growth. Our results indicate that B_12_ is released as a result of bacteriophage-mediated cell lysis of *Sulfitobacter* sp. M39, enabling the growth of *T. pseudonana* in co-culture and possibly other microbes in nature. Therefore, we propose that bacteriophage-mediated cell lysis is a key mechanism for the release of essential metabolites, including vitamins, and given the estimated bacteriophage infection rates in the ocean, it plays a crucial role in the B-vitamin cycle in the marine environment.

## Introduction

Vitamin B_12_ (cobalamin, B_12_) is an essential cofactor that functions in key metabolic processes and enzymatic reactions in both eukaryotes and prokaryotes [1, 2]. Whilst the majority of microorganisms require B_12_ [3], only a minor fraction of bacteria and archaea can *de novo* synthesize this coenzyme [4–10].

In pelagic marine ecosystems, B_12_ is mostly present at low concentrations, ranging from 90 pM to below 1 pM, the current detection limit [11–15]. About half of the cultivated marine microalgae are B_12_-auxotrophic, whilst only one quarter of cultivated marine bacteria possess the complete B_12_ biosynthetic pathway [7, 16]. The dependency of phototrophic eukaryotes on B_12_-prototrophic prokaryotes is further evident by the positive correlations between B_12_ biosynthesis potential in marine microbial communities and chlorophyll-*a* concentrations [17]. Additionally, data mining of bacterial metagenomes highlights the importance of metabolic cross-feeding, particularly of B-vitamins, as a strategy for most marine microbes to overcome metabolic dependencies [18–21].

In the recent past, several studies have described the provision of B_12_ by heterotrophic bacteria to auxotrophic phytoplankton as a mutualistic interaction [16, 22–26]. However, until now, a B_12_-export mechanism remains unknown [27]. Recently, it was demonstrated that one-third of all tested B_12_-prototrophic marine alphaproteobacteria did not voluntarily share this essential cofactor with adjacent phytoplankton, despite their reliance on organic carbon from the autotrophs [7, 26]. This observation implies that, in addition to the active provision of the cofactor, which is presumably enabled by an export system [27], the passive cofactor provision is likely to be an essential mechanism in nature, e.g. through cell lysis mediated by various factors [7, 28].

The two main causes for bacterial death in marine pelagic ecosystems are grazing by protists and viral infections [29, 30]. While grazing results mainly in the incorporation of bacterial biomass in protist biomass and oxidation of bacterially derived organic matter, viral infection leads to massive release of bacterially derived organic matter. Viruses in the surface seawater are present in quantities in the order of 10^10^ per litre, outnumbering bacteria by a factor of about 10 [29]. Bacteriophages, viruses that infect and lyse bacteria, are estimated to cause about 20%–40% of bacterial mortality in marine surface waters every day [30, 31], thereby freeing substantial amounts of organic matter into the surrounding water—a process known as viral shunt [32–35]. Moreover, it is believed that bacteriophage-mediated cell lysis leads to the release of essential intracellular metabolites that are vital for the metabolism of microbial communities [20, 36]. Additionally, there is evidence that bacterial cell lysis as a result of bacteriophage infection contributes to nitrogen cycling through the release of amino acids [37, 38] and proteinaceous material [39], and promotes the release of bioavailable iron for other microorganisms [40, 41]. In a recent study, it was shown that bacteriophage-mediated cell lysis enables the release of varying amounts of amino acids, depending on the bacteriophage strain infecting the same host [42]. Similarly, the induction of temperate phages with accompanying cell lysis can lead to the release of essential metabolites. It has recently been shown in a mutually dependent co-culture between two B_12_-auxotrophic bacteria that cross-feed B_12_-building blocks, that B_12_ is only released from a B_12_-retainer bacterium to a second bacterium through prophage-induced cell lysis [7].

Microbial communities are often intertwined in cross-feeding networks, where every species relies on metabolites synthesized and released by others and phage infection of one of the members can likely affect the dynamics of this network [43]. However, the impact of viral lysis on metabolite cross-feeding networks has not been widely studied [30, 44], especially the effect of viral lysis on B_12_ cross-feeding remains poorly understood.

In this study, we tested the hypothesis that bacteriophage infection of a B_12_-prototrophic, B_12_-retainer bacterium leads to the release of the cofactor and in turn promotes growth of a B_12_-auxotrophic microalgae. We assume that bacteriophages play an important role in the B_12_ cycling between prototrophic and auxotrophic microorganisms. We isolated bacteriophages specific for the bacterium *Sulfitobacter* sp. M39, which was identified as a B_12_-retainer in our previous study [26], and co-cultured this strain with the B_12_-auxotrophic diatom *Thalassiosira pseudonana* (CCMP 1335) in the presence and absence of the bacteriophages to monitor the variability in growth of the diatom.

## Materials and methods

### Bacteriophage isolation, purification, and preservation

A surface water sample was collected on 25 October 2023 to isolate bacteriophages from the shoreline of the North Sea in Neuharlingersiel (53.7039147 N, 7.7043601 E) at a water temperature of 12.5°C. The water sample was first filtered and then, 3 l were concentrated 10-fold to 300 ml using cross-flow filtration (Vivaflow® 50 R, PES membrane, 30 kDa MWCO HY, Sartorius). The derived seawater phage concentrate was again filtered (0.22 μm; Nalgene Rapid-Flow, PES membrane, Thermo Fisher Scientific) to remove potential bacterial contaminations prior to bacteriophage isolation. Bacteriophages were isolated using the spot test method, followed by three consecutive transfers onto fresh plates for purification. Glycerol stocks of the bacteriophages, specific to the host bacterium *Sulfitobacter* sp. M39, were prepared for long-term preservation. The isolation process was conducted as previously described [45], with minor modifications. Detailed method for the isolation of the bacteriophages is provided in Supplementary Text S1.

### Microscopic imaging of the bacteriophages

*Sulfitobacter* sp. M39 was grown in Marine Broth (MB)-medium at 20°C and 100 rpm. A cell pellet was collected in the mid-exponential growth phase by centrifugation (4000 *g*, 15 min, 4°C, Eppendorf 5430 R, Hamburg, Germany) and resuspended in 2 ml synchronized artificial seawater (syn-ASW) media [26]. Subsequently, the cell pellet was infected with the bacteriophages (500 μl) and incubated for an additional 24 h. The bacteriophage lysate was collected by centrifugation (4000 *g*, 15 min, 4°C) and filtration, using a 0.2 μm syringe filter (Rotilabo syringe filter, Carl Roth, Karlsruhe, Germany). For transmission electron microscopy (TEM) imaging, 30 μl of the bacteriophage lysate was loaded onto a carbon-coated grid (Formvarfilm, 200 mesh), adsorbed for five and dried for 10 min. The adsorption process was repeated three times before staining with 1% uranyl acetate for 45 s and subsequent washing with distilled water twice for 2 s. Grids were then air-dried for 15 min and afterwards visually analysed using TEM (Zeiss EM 900 N, Oberkochen, Germany). For fluorescence microscopy imaging, *Sulfitobacter* sp. M39 was grown in 10 ml MB-medium with and without the addition of the bacteriophages (100 μl from stock solution) as described above. Ten microlitre of each culture was spotted onto a glass slide, stained with SybrGreen I (120× final concentration, Invitrogen, Netherlands), and visualized under the microscope (Zeiss, objective lens Axiocam 705 colour, 100×). Images were taken using ZEN 3.8 Pro software.

### Genome sequencing, assembly, and annotation of the bacteriophages

An infection culture for the phage DNA extraction was prepared following the same procedure as described above for TEM imaging. A Qiagen DNeasy Blood & Tissue Kit was used for the DNA extraction, following the separation of the phage particles from bacterial cells prior to extraction. Whole genome sequencing of the bacteriophages was performed (Eurofins Genomics, Constance, Germany) using Illumina NovaSeq (PE 150 mode; Illumina, San Diego, USA), generating ~5 million read pairs per sample (2 × 150 bp paired-end reads), corresponding to an estimated genomic coverage depth of ~30 096×. For genome assembly, sequenced reads were mapped against the host genome (*Sulfitobacter* sp. M39) using bbmap.sh (sourceforge.net/projects/bbmap/) and the unmapped reads were extracted and assembled using metaspades.py [46]. Further information on the tools and methods used for assembly and annotation can be found in Supplementary material (Supplementary Text S2).

### Bacterial culture infected with bacteriophages

For preparing the bacteriophage stock for the main experiment, *Sulfitobacter* sp. M39 was grown in a mixture of half syn-ASW-medium supplemented with vitamins B_1_, B_2_, B_3_, B_5_, B_6_, and B_7_ each at 500 pM along with glucose, acetate, and glutamate, each at 4 mM C, and half (h)MB-medium, and was incubated overnight. The culture was then infected by adding bacteriophages to the bacterial host and incubated further for 24 h. Finally, bacteriophage particles were collected as described above.

In parallel, a preculture of *Sulfitobacter* sp. M39 grown in MB-media was collected in the late exponential growth phase, washed (twice, 4000 *g*, 5 min) with B_12_-free syn-ASW-media and subsequently used for the inoculation of the main experiment in B_12_-free syn-ASW-media. Cultures were incubated at 20°C, 100 rpm and growth was monitored by optical density (OD, 600 nm, Photolab, 7600 UV–VIS).

The growth experiment included two main treatments: one with the bacterial host culture alone and the second with the bacterial host culture and bacteriophages. Additionally, there were two control setups: one with only the bacteriophage lysate (no bacteria), and another with neither bacteriophage lysate nor bacteria. The cultivation volume for both treatments and controls was 250 ml and each was carried out in triplicate. Samples for bacterial cell count (BCC) and virus-like particle (VLP) enumeration were collected (every 1–2 h), fixed with glutaraldehyde (final concentration 1% for BCC and 0.5% for VLP) for 30 min at 4°C, and then stored at −20°C for BCC- and −80°C for VLP-samples until further analysis. After a drop in OD and the appearance of visible cell debris in the infected cultures, 100 ml of sample was collected from both treatments for extracellular B_12_ analysis. Samples were centrifuged (4000 *g*, 5 min, 4°C), filtered (0.2 μm) and then stored at −20°C until further analysis using liquid chromatography-mass spectrometry (LC–MS). To examine the possibility of B_12_ uptake by the B_12_-prototrophic bacterium *Sulfitobacter* sp. M39, cultures were again grown in syn-ASW-medium in triplicate with and without the addition of B_12_ (100 pM). Samples for extracellular B_12_ analysis were collected and stored as described above.

### Co-culture of *T. Pseudonana* and *Sulfitobacter* sp. M39 with and without bacteriophages

A B_12_-depleted preculture of the axenic *T. pseudonana* culture (CCMP 1335, kindly provided by Dr. Nicole Poulsen, Technical University of Dresden, Germany) was prepared by first growing the diatom in NEPCC media (Supplementary Table S1). Subsequently, it was transferred (twice) to B_12_-free syn-ASW media and in the second transfer, the diatom preculture was supplemented with 10 pM B_12_ to ensure a minimum growth. *Sulfitobacter* sp. M39, a B_12_-retainer [26], was cultured in MB-media, harvested in the late exponential growth phase and washed twice with B_12_-free syn-ASW-media prior to inoculation as described above. The co-culture experiment consisted of two main treatments, each ran in triplicates. First, the diatom was co-cultured with *Sulfitobacter* sp. M39 without additional supplements, to determine whether the bacterial strain voluntarily supplies B_12_ to the auxotrophic diatom. Secondly, the diatom was grown with *Sulfitobacter* sp. M39 and spiked with bacteriophage lysate (400 μl, after 96 h), to test whether bacteriophage-mediated cell lysis enables diatom growth. Alongside the co-culture experiment, the diatom was grown with (100 pM) and without addition of B_12_, serving as negative and positive controls. For all co-culture treatments, the initial bacterial inoculum was determined to be ~1 million cells/ml (based on flow cytometric counting) and the initial diatom cell inoculum was determined to be ~4000 cells/ml (based on microscopic counting). All diatom cultures were illuminated at 70 μE m^−2^ s^−1^ and incubated at 20°C with a 12:12 h light–dark cycle (RUMED, Rubarth Apparate GmbH, Laatzen, Germany). Growth of *T. pseudonana* was monitored throughout the experiment by measuring relative chlorophyll-*a* fluorescence (RF; TD 700 fluorometer, Turner Designs, CA, USA) with excitation at 340–500 nm and emission at 680 nm. Samples for diatom cell counts, BCC and VLP were collected periodically. BCC and VLP samples were fixed and stored as described above. Diatom cell count samples were fixed with Lugol (final concentrations of 0.15% iodine and 0.29% potassium iodide) and stored at 4°C until further analysis.

### Enumeration of bacterial cells, VLPs, and diatom cells

BCC and VLP samples were enumerated by flow cytometry (BD Accuri Plus, BD biosciences, Franklin Lakes, NJ, USA). BCC samples from the bacteria-phage growth experiment were first diluted with sterile TE-buffer according to the bacterial count, stained with SybrGreen I (10× final concentration), and enumerated as described elsewhere [47]. An additional detachment method was performed for the BCC samples of the bacteria-diatom co-culture experiment as described elsewhere [26, 48]. The VLP samples were first diluted with sterile, 0.2 μm filtered TE buffer according to the bacterial and VLP load, stained with SybrGreen I (1:20 000 final dilution), and enumerated following a method described elsewhere [49, 50]. All BCC samples were measured for 2 min (threshold FL1-H 800, slow fluidics), whilst VLP samples were measured for 1 min (threshold FL1-H 250, slow fluidics). Lugol fixed diatom samples from the co-culture were loaded on a haemocytometer and enumerated by microscopy (AXIO, Lab.A1, objective lens Carl Zeiss, 40×).

### Extracellular B_12_ measurement

For extracellular B_12_ analysis, samples were adjusted to pH 6, concentrated using solid phase extraction (Bond Elut PPL, 1 g, Agilent) and eluted with methanol [51]. A nitrogen stream was applied to evaporate the solvent and the residues were then dissolved in 100 μl ultrapure water for ultra-high performance liquid chromatography (UHPLC) separation with a Vanquish Flex (Thermo Fisher Scientific, MA, USA) on a Kinetex Evo C18 column (100 × 2.1 mm, 2.6 μm pore size, Phenomenex, Torrance, CA, USA). Ten millimolar ammonium formate (pH 6.0) (A) and acetonitrile (B) were used with the following solvent gradient: 0–6 min 100%–75% A; 6–7 min 75%–0% A; 7–8.5 min 0% A; 8.5–9 min 0%–100% A; 9–10 min 100% A. An Orbitrap Fusion mass spectrometer (Thermo Fisher Scientific, MA, USA) in full scan mode was used for identification of the individual extracellular B_12_ forms based on their exact masses [52], which can be found in Supplementary Table S2. Respective analytes were quantified by external calibration, using commercially available standard compounds and were summarized as total B_12_.

## Results

### Bacteriophage isolation, verification, and genome sequencing

**Figure 1:**
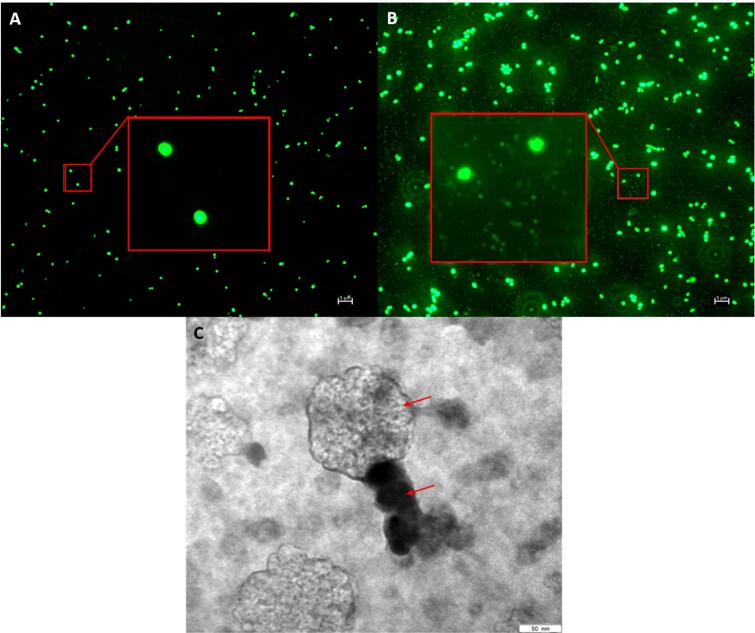
Microcopic images of *Sulfitobacter* sp. M39 and bacteriophages. Fluorescence microscopic images of the bacterial host *Sulfitobacter* sp. M39 without (A) and with (B) the bacteriophages (M39 + M39B, stained with SybrGreen I, 100× objective lens) and (C) transmission electron micrograph (stained with uranyl acetate, 250 000× magnification) of the bacteriophages. Insets in (A) and (B) show 500× magnified images. The arrows indicate the tail (dark) and head of the bacteriophage.

The spot assay revealed clear plaques formed on the soft agar plates containing the bacterial host *Sulfitobacter* sp. M39. After conducting three-times a dilution plating from individual plaques, we prepared our bacteriophage lysate stock solution (Supplementary Fig. S1). Bacteriophage presence was further confirmed by fluorescence microscopy (Fig. 1A and B) and TEM imaging (Fig. 1C, Supplementary Fig. S2). Bioinformatic analysis following the genome sequencing recovered the presence of two phage sequences, both containing double stranded DNA and affiliated to the class *Caudoviricetes*, with genome sizes of 49 844 and 43 809-bp, respectively. Both these assembled phages contained direct terminal repeats (DTRs), confirming they are not prophages. Additionally, all reads mapping to the host genome were removed prior to the assembly and phage identification steps, which is ensuring that these two phages originated from the isolation process in this study. The coverage analysis indicated the 49 844-bp phage as the lytic phage, with an average coverage of 2329 reads per position compared to 14.2 reads per position for the 43 809-bp phage. No confirmation that the 43 809-bp phage is a temperate phage was detected. Whilst the lower coverage plus DTRs of this phage genome hints to a pseudolysogeny or episomal growth strategy for the 43 809-bp phage, at the moment we do not have enough empirical proof to conclude that. Therefore, in the following experiments, we refer to these two phages as bacteriophage M39 and bacteriophage M39B (herein bacteriophages). Both bacteriophage genome sequences were deposited to NCBI GenBank database under the accession number PQ900653 for bacteriophage M39 and PV985214 for bacteriophage M39B. Further information on the bacteriophage genome annotation can be found in Supplementary material (Supplementary Data S1, Supplementary Data S2, and Supplementary Text S2).

### Growth of *Sulfitobacter* sp. M39 infected by bacteriophages

**Figure 2:**
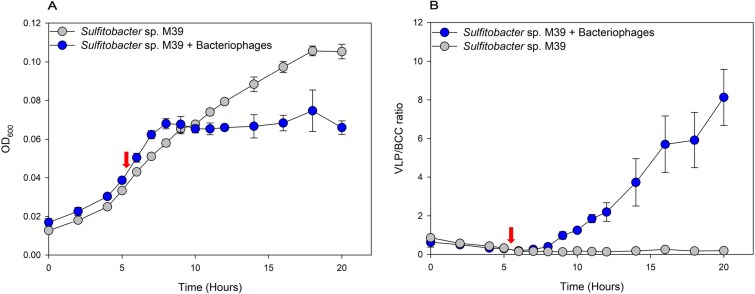
Growth curve of *Sulfitobacter* sp. M39 and VLP:Bacteria ratio over time. Effect of bacteriophage infection on growth of *Sulfitobacter* sp. M39. (A) Shows the growth over time (20 h) of *Sulfitobacter* sp. M39 with (blue circle) and without (grey circle) addition of bacteriophages (M39 + M39B) monitored by OD. (B) Shows the VLP/BCC ratios of *Sulfitobacter* sp. M39 with (blue circle) and without (grey circle) addition of the bacteriophages over the course of the bacterial growth curve. Error bars indicate the standard deviation of triplicates and arrows indicate the time of bacteriophage lysate addition.

To observe the effect of a bacteriophage infection on growth of the bacterial host, bacteriophage lysate (bacteriophage M39 and M39B) was added to *Sulfitobacter* sp. M39 cultures in the early exponential phase (OD ~ 0.04) after 5 h of inoculation. In both treatments, with and without the bacteriophage added, the OD continued to increase over a period of 2.5 h, and in the bacteriophage treated culture, an even steeper increase was observed in measured OD. After 10 h, a reduction in the OD was observed in the bacteriophage-treated culture and then stagnated at around 0.065 (OD), whilst the bacterial control continued to grow exponentially to a maximum OD of 0.105 before entering the stationary phase at hour 18 (Fig. 2A). An initial increase in the VLP/BCC ratio, determined from flow cytometric counts, in the phage-treated culture was observed after 8 h of incubation and 2.5 h after adding bacteriophage particles (Fig. 2B) and continued to rise until it reached to a ratio of 8.1 ± 1.5. In contrast, the VLP/BCC ratio in the bacterial control remained low throughout the entire bacterial growth curve, with the highest ratio reached at 0.3 ± 0.01 (Fig. 2B).

### Growth of *T. Pseudonana* in co-culture with *Sulfitobacter* sp. M39 and the bacteriophages

**Figure 3:**
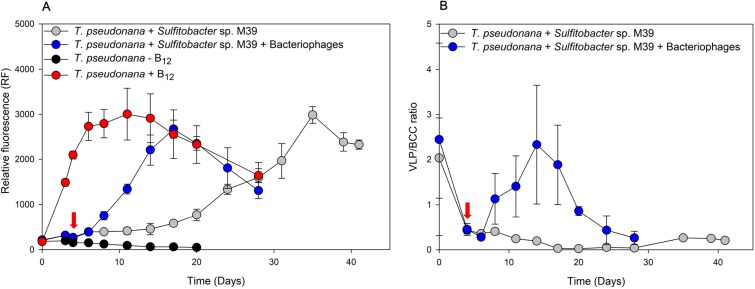
Growth of *Thalassiosira pseudonana* co-cultured with *Sulfitobacter* sp. M39 and with and without bacteriophages and VLP:Bacteria ratio over time. Growth of *T. pseudonana* in co-culture with *Sulfitobacter* sp. M39 and bacteriophages (M39 + M39B) over time. (A) Shows the growth of *T. Pseudonana* over time (42 days) with *Sulfitobacter* sp. M39 and with (blue circle) and without (grey circle) addition of bacteriophages monitored by measuring RF (chlorophyll a; excitation: 340–500 nm, emission: 680 nm). Additionally, growth of the diatom without (black circle) and with (red circle) addition of B12 are shown. (B) Shows the VLP/BCC ratios of *Sulfitobacter* sp. M39 with (blue circle) and without (grey circle) addition of the bacteriophages over time in diatom-bacteria co-culture. Error bars indicate the standard deviation of triplicates and arrow indicates the time of bacteriophage lysate addition.

To investigate whether bacteriophage-mediated cell lysis of the B_12_-retainer *Sulfitobacter* sp. M39 [26] leads to growth of the B_12_-auxotrophic diatom *T. pseudonana*, both were grown in co-culture in presence and absence of the host-specific bacteriophages. Before adding the bacteriophage lysate, relative fluorescence (RF) reflecting diatom growth, remained below 300 in both treatments. After bacteriophage lysate addition on day 4, RF of the diatom in the phage-treated culture increased exponentially from day 8 onwards and reached a maximum RF of 2673 ± 199 on day 17. In contrast, RF of the diatom in the diatom-bacterial control treatment (no bacteriophage lysate added), remained low until day 17 and afterwards increased continuously, however, with a higher doubling time (Supplementary Table S3), until day 35 (Fig. 3A). The diatom positive control, solely spiked with 100 pM B_12_, started growing from day 3 onwards and reached its maximum RF of 3003 ± 571 at day 11. In the negative control, no growth of the diatom was observed. The VLP/BCC ratio of the phage-treated diatom-bacteria culture showed a steep increase from day 8 onwards and continued until day 14, reaching a maximum ratio of 2.3 ± 1.3. The increase of the VLP/BCC ratio coincides with the initiation of diatom growth of the phage-treated co-culture. In contrast, the VLP/BCC ratio of the bacterial control treatment (no bacteriophage addition), remained low throughout the exponential and stationary growth phase of the diatom with the highest ratio reaching 0.4 ± 0.07 (Fig. 3B).

### Extracellular B_12_ detection

Extracellular B_12_ was measured in mono-cultures of *Sulfitobacter* sp. M39, spiked with and without the host-specific bacteriophages, to confirm the release of B_12_ as a result of phage-mediated cell lysis. No extracellular B_12_ was detected in the bacterial control treatment (no phage addition), whilst we could detect 10.4 ± 0.8 pM B_12_ in the phage-treated culture (Supplementary Table S4). To assess whether even after being a prototroph, *Sulfitobacter* sp. M39 takes up extracellular B_12_, we set up another experiment by growing this bacterium with and without the addition of 100 pM B_12_ and measured the extracellular B_12_ concentration. Again, no B_12_ was detected in B_12_-free bacterial culture, however, despite adding 100 pM B_12_ in the second treatment, we detected only 45.6 ± 0.4 pM B_12_ (Supplementary Table S5).

## Discussion

### Factors influencing B_12_ release by *Sulfitobacter* sp. M39

*Sulfitobacter* sp. M39 was identified as B_12_-retainer in a previous study, where it was grown together with *T. pseudonana* and growth was monitored for 25 days, with a maximum RF of 876 measured after 7 days and declining thereafter [26]. In this experiment, we observed a slight increase in diatom cell density after about 3 weeks of incubation when co-cultivated with *Sulfitobacter* sp. M39 without B_12_ addition. We then continued monitoring growth over 41 days and recorded a steady, albeit with a significantly higher doubling time compared to the bacteriophage addition experiment (Fig. 3A and Supplementary Table S3).

Two adjustments were made here in comparison to the previous study [26]. First, in this study, we used a new axenic *T. pseudonana* culture, likely undergoing a different asexual growth phase, featuring larger cells compared to our previous culture. During their asexual life cycle, centric diatoms shrink in cell size due to their bipartite silica cell wall. Once a critical cell size reduction is reached, diatoms enter a sexual phase under sufficient environmental conditions to recover an optimal cell size to re-enter the asexual size reduction cycle [53–55]. Apart from varying cell sizes, a lower maximum fluorescence was detected in the former culture and twice as many cells were observed in the former culture compared to the current culture to achieve the same fluorescence. Thus, we suppose that the *T. pseudonana* culture used in Sultana *et al*. [26]*,* characterized by smaller cell size, had a shorter life cycle. We speculate that B_12_ can also be released as a result of natural bacterial cell death and the resulting cell lysis, which in case of the former co-culture, likely occurred after all diatom cells had died. In contrast, we suspect that the natural bacterial death in the current culture occurred before all diatom cells died, allowing a late but gradual growth of the diatom culture. Secondly, when we identified *Sulfitobacter* sp. M39 as B_12_-retainer, we inoculated with ~500 000 cells/ml [26], whereas here, we spiked the diatom culture with ~1 million cells/ml. This time, we doubled the bacterial inoculum to ensure a sufficient cell number of *Sulfitobacter* sp. M39 for the bacteriophage infection and subsequent cell lysis, so that enough B_12_ would be released to observe an increase in algal growth. Due to the elevated bacterial count in culture, more B_12_ was likely released as a result of natural cell lysis, which could have additionally led to a longer survival of the diatom cells.

### B_12_ utilization by the B_12_-prototrophic bacterium *Sulfitobacter* sp. M39

To provide analytical evidence that B_12_ is released as a result of bacteriophage-mediated cell lysis, we grew *Sulfitobacter* sp. M39 in mono-culture with and without addition of bacteriophages (Bacteriophage M39 and M39B) and determined extracellular B_12_ concentrations. We detected 10.4 ± 0.8 pM extracellular B_12_ shortly after the occurrence of cell lysis in the bacteriophage-treated culture and none in the bacterial control culture (Supplementary Table S4). However, the high RF of the diatom in co-culture with *Sulfitobacter* sp. M39 and bacteriophages, compared to a previously conducted bioassay [26], suggests that the concentration of extracellular B_12_ released by phage-mediated cell lysis was most likely higher compared to our findings in mono-culture (Fig. 3). Thus, we speculated whether a fraction of B_12_ released due to the phage-mediated lysis may have been rapidly taken up by remaining, not infected, *Sulfitobacter* sp. M39 cells. To test our assumption, we conducted another experiment in which we grew *Sulfitobacter* sp. M39 with and without 100 pM B_12_ in mono-culture and collected samples for extracellular B_12_ measurements in the late exponential growth phase. This time, we detected 45.6 ± 0.4 pM B_12_, indicating that more than 50% of the supplied B_12_ was absorbed or no longer present in its initial state (Supplementary Table S5). One possible explanation here is that the molecular integrity of the supplied cyanocobalamin (CN-B_12_) was affected by abiotic factors. In particular, photodegradation can significantly reduce the presence of CN-B_12_ within a few hours [56]. However, our mono-culture was incubated in the dark at 20°C and was therefore not exposed to UV radiation. Also, known data indicate that only temperatures of 37°C in salt water after up to 9 weeks have a negative effect on the stability of CN-B_12_ [57]. Our data therefore suggest that *Sulfitobacter* sp. M39, even though being a B_12_-prototroph, utilizes extracellular B_12_. *Sulfitobacter* sp. M39 also encodes *btuD*, a gene essential for B_12_ uptake, which further supports our assumption. Our conclusion is consistent with recent findings that *Pseudomonas aeruginosa* takes up extracellular B_12_ under various environmental conditions despite possessing the complete B_12_ biosynthetic pathway [58]. This bacterial adaptation, reducing own biosynthetic efforts and instead taking up available metabolites from the environment, has also been shown for other metabolites. This way, metabolic costs are reduced, thus optimizing the use of resources, and increasing biomass production [59–61]. Especially since B_12_ biosynthesis is a metabolically expensive process, requiring multiple enzymatic reactions [3, 4]. Our findings raise the question of the extent to which B_12_-prototrophic bacteria in marine and other ecosystems can be considered B_12_ producers. Thus, deciphering B_12_ cross-feeding networks within complex microbial communities becomes even more complicated [21, 62].

### Bacteriophage-mediated cell lysis and B_12_ release

In our study, we hypothesized that bacteriophage-mediated cell lysis of B_12_-prototrophic, yet B_12_-retainer bacteria can make this essential co-factor available for auxotrophic microbes. To confirm this hypothesis, we isolated host-specific bacteriophages, that we then added to a *Sulfitobacter* sp. M39 mono-culture and co-culture, containing the B_12_-auxotrophic diatom *T. pseudonana*. Shortly after adding the bacteriophages to the mono-culture, we observed a bacterial growth stagnation coinciding with an VLP/BCC ratio increase, indicating a lytic life cycle of the bacteriophages in *Sulfitobacter* sp. M39 (Fig. 2 and Supplementary Fig. S3A). Similarly, when infecting the bacterial host with the bacteriophages in co-culture, growth of the bacterium halted, coinciding with a rapid increase in growth of the B_12_-auxotrophic diatom and a steep increase in the VLP/BCC ratio (Fig. 3 and Supplementary Fig. S3B). Bacteriophage M39, which accounted for 99.6% of the total bacteriophage coverage, encodes hydrolase and peptidase M15 superfamily proteins (Supplementary Data S1), which are hydrolytic enzymes predicted to degrade the peptidoglycan layer of the bacterial cell wall, resulting in cell lysis [63, 64]. Additionally, it encodes an iron–sulphur cluster binding protein, predicted to be a component of phage-tails, that is involved in host cell adsorption and genome injection [65]. These features are likely crucial for enabling a bacteriophage to establish an effective infection cycle in its host bacterium. We were able to rule out the possibility that release of methionine from the lysed bacterial cells accounts for the observed diatom growth, as B_12_ dependency of *T. pseudonana* is exclusively expressed through the B_12_-dependent methionine synthase (*metH*) [16, 66], by previously demonstrating that the addition of methionine alone does not stimulate growth of *T. pseudonana* [26]. Hence, we conclude that the lytic life cycle of the bacteriophages in *Sulfitobacter* sp. M39 and its subsequent cell lysis results in the release of B_12_, thereby promoting growth of the B_12_-auxotrophic diatom. Similar to our findings, previous studies have shown the influence that bacteriophages or viruses can have on the release of metabolites. Experimentally, it has been shown that essential nutrients and organic compounds such as amino acids are released upon virus-mediated cell lysis [38, 40, 41, 67]. Furthermore, not only important organic compounds and inorganic nutrients are released, but a virus or bacteriophage infection can also lead to an altered metabolism of the host, which can result in a modified metabolite synthesis [37]. Notably, Pherribo and Taga [42] showed that the infection of different bacteriophages within the same bacterial host strain resulted in different amino acid compositions in the cell lysate. At present, whether B_12_ synthesis efficacy or dissolved B_12_ quantity in bacteriophage-mediated cell lysate differ amongst varying bacteriophage infections remains an open question.

### Ecological impact of bacteriophage-mediated cell lysis on essential metabolite cycling

B_12_ is an essential cofactor for vital metabolic processes in both prokaryotes and eukaryotes. Only a minor fraction of bacteria and archaea are known to produce B_12_ [5]. In marine environments, microbial B_12_-consumers outnumber B_12_-producers by far, thus limiting the growth of B_12_-auxotrophs [14, 68–70]. This disparity between production and availability underscores the importance of a B_12_ release mechanism. It has long been assumed that the coenzyme is secreted by B_12_-producers, but we have shown that only some bacteria, about one-third in our study, actually release the coenzyme efficiently to support the growth of a phototrophic B_12_-auxotroph [7, 26]. This finding highlights the importance of a passive metabolite release mechanism. Moreover, considerable differences in intra- and extracellular B_12_ concentrations have been reported for a variety of B_12_-provider strains, whereby a passive release mechanism would set significantly more B_12_ free [12, 26, 52]. Ultimately, every B_12_ prototroph will serve as a B_12_ source upon cell lysis, regardless of being a B_12_-provider or a B_12_-retainer [27, 71].

Here, we demonstrate a mechanism for passive B_12_ release by means of bacteriophage-mediated cell lysis. Viruses are considered to be the most abundant and dynamic entities of life in the aquatic environment, with an approximate abundance of about 10^10^ viruses per litre of surface seawater [29]. In the ocean, bacteriophages account for up to 40% of the daily mortality of bacterioplankton [72], releasing substantial amounts of dissolved organic matter, likely including essential metabolites [29]. This process is known as “viral shunt,” where bacteriophages make the cellular biomass available in the form of dissolved and particulate organic matter for further utilization by the microbial community [33, 34, 73]. In our artificial experiment, the growth of the heterotrophic bacterium was tied to the release of algae-derived organic substances. However, algae growth was first enabled by the release of B_12_ through bacteriophage-mediated cell lysis, whereby the bacteriophage ultimately promoted the growth of its bacterial host. Despite the importance of bacteriophages shaping microbial population dynamics and driving global geochemical cycles, their impact on metabolite cross-feeding networks remains largely unknown [30, 32, 44, 74, 75]. Our data provide experimental evidence of a bacteriophage infection and consequent cell lysis of a B_12_-prototrophic, B_12_-retainer bacterium and the resulting release of B_12_, leading to the growth of a B_12_-auxotrophic diatom, *T. pseudonana* (Fig. 3). In nature, highly abundant bacteria are often targeted by bacteriophage infections, which is commonly known as the “Killing the Winner” hypothesis [76, 77]. Our laboratory-based results suggest that local metabolite hotspots can form in the wake of bacteriophage infections, which can then significantly promote the growth of auxotrophs, a factor that could favour bacterio- or phytoplankton succession in nature [78]. This might further contribute to the evolution of auxotrophies and formation of microbial network of interdependencies [20]. Finally, considering the high frequency of bacteriophage-mediated cell lysis in the vast ocean, cell lysates can serve as a tremendous source of crucial metabolites [79] and thus also contribute substantially to the B-vitamin cycle [27, 71].

## Supplementary Material

Supplementary_Data_1_ycaf136

Supplementary_Data_2_ycaf136

Supplementary_Information_All_in_one_ycaf136

## Data Availability

The datasets generated during the current study are available from the corresponding author on reasonable request.
