# Supplementary material for: Vitamin B12 release through bacteriophage-mediated cell lysis of the marine bacterium Sulfitobacter sp. M39
Source: ISME Commun. 2025 Aug 29;5(1):ycaf136. doi: 10.1093/ismeco/ycaf136 (PMC12456174; doi:10.1093/ismeco/ycaf136)
Supplement: Supplementary_Information_All_in_one_ycaf136 [file supplementary_information_all_in_one_ycaf136.pdf]

Supplementary Information:

Vitamin B_{12} release through bacteriophage-mediated cell lysis of the marine bacterium *Sulfitobacter* sp. M39

Figure and table legends

Supplementary information

Table S1: Composition of the medium NEPCC used, to promote the growth of *T. pseudonana*.

Table S2: Molecular Ion masses (*m/z*) for identification of analytes after full scan measurement on an Orbitrap Fusion mass spectrometer (Thermo Fisher Scientific).

Table S3: Doubling time of *T. pseudonana* in co-culture with *Sulfitobacter* sp. M39, with and without addition of bacteriophages (M39 + M39B), and in mono-culture with addition of B_{12}.

Table S4: Extracellular B_{12} concentration measured from B_{12}-retainer strain *Sulfitobacter* sp. M39 in presence and absence of the bacteriophages (M39 + M39B).

Table S5: Extracellular B_{12} concentration measured from B_{12}-retainer strain *Sulfitobacter* sp. M39 supplemented with 100 pM B_{12} and no B_{12}.

Supplementary Data 1: Additional information about bacteriophage M39 annotated genes.

Supplementary Data 2: Additional information about bacteriophage M39B annotated genes.

Supplementary Text 1: Additional information about bacteriophage isolation, purification, and preservation.

Supplementary Text 2: Additional information about tools used for bacteriophage genome annotation and significant features of the bacteriophages.

Figure S1: Isolation of bacteriophages from the North Sea water using spot test method (A), followed by purification through three successive transfers onto fresh plates containing host bacterial culture (B).

Figure S2: Transmission electron micrograph (stained with uranyl acetate, 250 000x magnification) of the bacteriophages. Red arrows indicate the tail (dark) and head of the bacteriophages.

Figure S3: *Sulfitobacter* sp. M39 cell counts without and with addition of bacteriophages, in absence (A) and presence of *Thalassiosira pseudonana* (B). Error bars indicate the standard deviation of triplicates and red arrows indicate the time of bacteriophage lysate addition.

Table S1: Composition of the medium NEPCC used, to promote the growth of *T. pseudonana*.

	1L
NaCl	20.8 g
$MgCl_2$ x $6H_2O$	9.6 g
Na_2SO_4	3.5 g
1M $CaCl_2$	9 ml
KCl	0.6 g

Autoclave the media, add the following supplement and enrichment solutions, and adjust the pH to 8.0 – 8.2 with 1M HCl

Respective volume of enrichment solutions:

No: 1-5 1 ml/L, No: 6 100 µl/L

Enrichment solution recipes:

**Enrichment solutions**	
1. $NaNO_3$	47 g/L (533mM)
2. $SrCl_2$ x $6H_2O$	21 g/L
3. Na_2SiO_3 x $9H_2O$	30 g/L (105mM)
4. Na_2EDTA x $2H_2O$	1.86 g
$ZnCl_2$	32.7 mg
$CoCl_2$ x $6H_2O$	20.2 mg
Na_2MoO_4 x $2H_2O$	126 mg
$MnCl_2$ x $4H_2O$	475 mg
Dissolve in 800ml Milli-Q water, fill up to 1L, and adjust to pH 6 with 1M NaOH	
5. H_3BO_3	25 g/L
6. Na_2SeO_3 x $5H_2O$	1.73 g/L

Respective volume of supplement solutions:

I-III each 1 ml/L

Supplement solution recipes:

**Supplement I**		
A: dissolve in 450 ml Milli-Q	Na_2EDTA x $2H_2O$	3.72 g
	$FeCl_3$	1.76 g
First dissolve the EDTA and add slowly the $FeCl_3$. Adjust the pH to 6 and let the solution stir until it is clear.		
B: dissolve in 450 ml Milli-Q	NaF	3 g
		85 g
KBr		
Combine solution A + B and fill up to 1 L with Milli-Q.		

**Supplement II**	
Na_2-Glycerophosphate	6 g
Dissolved in 1L saturated solution of $NaHCO_3$ (approximately 100 g/L)	

**Supplement III**	
Thiamin HCl	200 mg/L
Vitamin B_{12}	4 mg/L
Biotin	2 mg/L

Table S2: Molecular Ion masses (*m/z*) for identification of analytes after full scan measurement on an Orbitrap Fusion mass spectrometer (Thermo Fisher Scientific).

Analyte	Chemical Formula	Molecular Ion Mass (*m/z*)
Cyanocobalamin (CB_{12})	$C_{63}H_{88}CoN_{14}O_{14}P$	678.2910
Adenosylcobalamin (AB_{12})	$C_{72}H_{100}CoN_{18}O_{17}P$	790.3364
α-Ribazole (α-Rib)	$C_{14}H_{18}N_2O_4$	279.1339
5,6-Dimethylbenzimidazole (DMB)	$C_9H_{10}N_2$	147.0917

Table S3: Doubling time of *T. pseudonana* in co-culture with *Sulfitobacter* sp. M39, with and without addition of bacteriophages (M39 + M39B), and in mono-culture with addition of B_{12}.

**Sample**	**Doubling time of *T. pseudonana* (Day)**
T. pseudonana + *Sulfitobacter* sp. M39	9.6 ± 0.6
T. pseudonana + *Sulfitobacter* sp. M39 + bacteriophages	4.1 ± 0.2
T. pseudonana + B_{12}	3.2 ± 1.3

Table S4: Extracellular B_{12} concentration measured from B_{12}-retainer strain *Sulfitobacter* sp. M39 in presence and absence of the bacteriophages (M39 + M39B).

**Sample**	**Sample collection method**	**Extracellular B_{12} concentration (pM)**
Sulfitobacter sp. M39	Filtration	Not detected
Sulfitobacter sp. M39 + bacteriophages	Filtration	10.4 ± 0.8

Table S5: Extracellular B_{12} concentration measured from B_{12}-retainer strain *Sulfitobacter* sp. M39 supplemented with 100 pM B_{12} and no B_{12}.

Sample	Sample collection method	Extracellular B_{12} concentration (pM)
Sulfitobacter sp. M39	Filtration	Not detected
Sulfitobacter sp. M39 + 100 pM B_{12}	Filtration	45.6 ± 0.4

Supplementary Text 1: Additional information about bacteriophage isolation, purification, and preservation.

Phage enrichment culture

First, a pre-culture of *Sulfitobacter* sp. M39 was grown in marine broth (MB)-media at 20 °C, 100 rpm (Labwit scientific & Zhicheng, Australia). Growth was monitored by measuring the optical density (OD_{600}, Photolab, 7600 UV-VIS) over time. In order to obtain a M39 host-specific phage enrichment culture, one part of media (Tenfold concentrated MB-media containing ten times the amount of peptone and yeast extract in comparison with basic MB-media) was mixed with nine parts of the seawater phage concentrate. This media - seawater phage concentrate mix was then inoculated with the host bacteria. In addition, two control cultures were incubated: a positive control containing MB instead of seawater, in order to monitor host growth and a negative control containing medium and seawater, but no host inoculum to monitor any growth of bacterial contaminants in the seawater phage concentrate. Bacterial growth was monitored measuring OD_{600}. The enrichment cultures were incubated overnight at 20 °C, 100 rpm. On the following day, bacterial cells were removed by centrifugation (4000 g, 15 mins, 4 °C, Eppendorf 5430 R, Hamburg, Germany) and further filtered using 0.2 µm syringe filter (Rotilabo syringe filter, Carl Roth, Karlsruhe, Germany) to collect the enriched phage fraction.

Spot assay and phage purification

Spot assays were performed to visualize the phage infection on agar plates. For this purpose, hMB (50% MB) plates with 1.8% agar was used as base layer and molten hMB with 0.6% agar (cooled to about 50 °C) was used as top layer. Then 280 µl of exponentially growing host bacterial culture and 3 ml of soft agar were added to the center of the plate and mixed well to achieve an even distribution of the host bacteria. After the plates were completely dry, five drops of the phage lysate were added to five different spots on duplicate plates. Alongside, two experimental controls were considered. In the first control, only molten soft agar was added to monitor any bacterial contamination of the media. In a second control, only the host bacterial culture and molten agar, but no phage lysate, were added to test for any phage contamination in the host bacterial culture. The plates were placed upright until completely dry and incubated as such at 20 °C for 24 hours. Once plaques were visible on the plates, individual plaques were transferred to new plates (as mentioned before) in order to isolate pure phages. Then, 280 µl of host bacterial culture and 3 ml of molten soft agar were added to a base plate and

mixed vigorously to thoroughly homogenize and distribute the mix. While the soft agar was still liquid, sample material was taken from individual plaques using a sterile pipette tip and streaked through the molten agar to obtain isolated phage. Subsequently, plates were incubated at 20 °C. We repeated this procedure three times in order to maintain pure phages.

Preservation of phages

After three transfers of the plaques, glycerol stocks were prepared for long term preservation. For this purpose, an infection culture was prepared by infecting 10 ml of exponentially growing host bacterial culture (in MB) by adding a single plaque from the last plaque transfer plate. The culture was then incubated overnight at 20 °C, 100 rpm. The following day, phage lysate was collected as described above. The isolated phage was preserved in two different glycerol stocks at -80 ° C. One glycerol stock was prepared with 250 µl host bacterial cultures, 250 µl phage lysate and 500 µl glycerol mixture (50 % hMB + 50 % glycerol) and the second with 500 µl phage lysate and 500 µl glycerol mixture.

Supplementary Text 2: Additional information about tools used for bacteriophage genome annotation and significant features of the bacteriophages.

Tools used for bacteriophage genome annotation:

Assembled contigs were used as input to run geNomad for identification of mobile genetic elements using default parameters [1]. Recovered phage sequences were used for gene prediction using prodigal [2] and presence of key phage genes was manually validated. Annotation was performed using NCBI conserved domain [3] and eggnog-mapper (v.2.1.12) [4] using database version 5 [5]. Abundance of the recovered phage genome in sequenced reads was calculated using coverM (https://github.com/wwood/CoverM/blob/main/CITATION.cff) and represented as average number of aligned reads overlapping each position on the contig.

Significant features of bacteriophage genomes:

In the sequenced cell free lysate two phage sequences were recovered, both containing double stranded DNA phage affiliated to *Caudoviricetes*. These two phages have a genome size of 49 844 and 43 809 bp and both contain direct terminal repeat. A total of 73 and 55 coding sequences was predicted in these phage genomes respectively. The 49 844-phage genome contains genes annotated as terminase small subunit and tape measure protein and the 43 809-phage encodes phage tail protein among hallmark phage genes.

References:

1. Camargo AP, Roux S, Schulz F, Babinski M, Xu Y, Hu B, et al. Identification of mobile genetic elements with geNomad. *Nat Biotechnol* 2024;**42**:1303–12.
2. Hyatt D, Chen GL, LoCascio PF, Land ML, Larimer FW, Hauser LJ. Prodigal: prokaryotic gene recognition and translation initiation site identification. *BMC Bioinformatics*. 2010, 11:119
3. Wang J, Chitsaz F, Derbyshire MK, Gonzales NR, Gwadz M, Lu S, et al. The conserved domain database in 2023. *Nucleic Acids Res* 2023;**51**:D384–8.
4. Cantalapiedra CP, Hernandez-Plaza A, Letunic I, Bork P, Huerta-Cepas J. eggNOG-mapper v2: Functional Annotation, Orthology Assignments, and Domain Prediction at the Metagenomic Scale. *Mol Biol Evol* 2021;**38**:5825–9.
5. Huerta-Cepas J, Szklarczyk D, Heller D, Hernández-Plaza A, Forslund SK, et al. EggNOG 5.0: A hierarchical, functionally and phylogenetically annotated orthology resource based on 5090 organisms and 2502 viruses. *Nucleic Acids Res* 2019;**47**:D309–14.

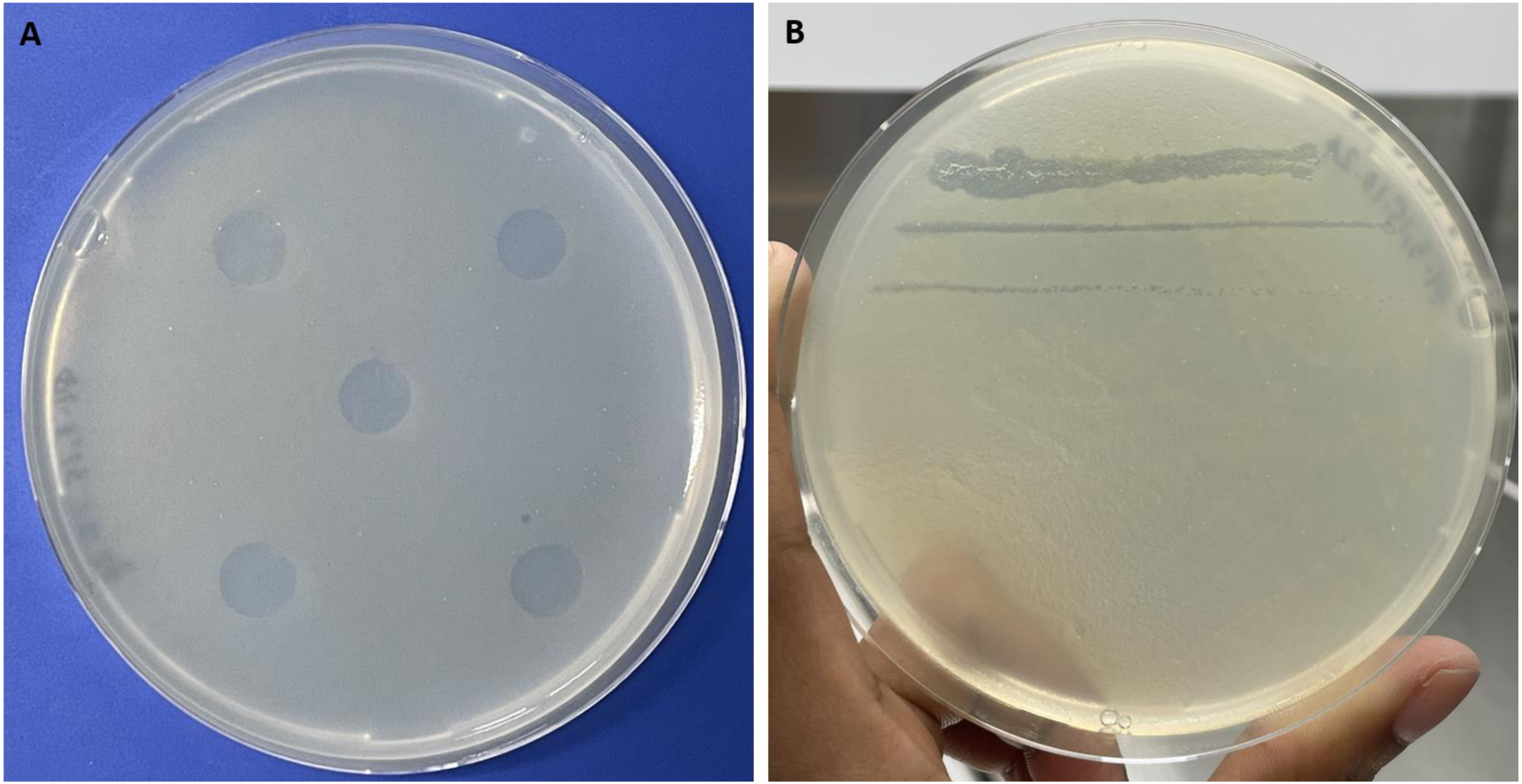

Supplementary Figure S1: Isolation of bacteriophages from the North Sea water using spot test method (A), followed by purification through three successive transfers onto fresh plates containing host bacterial culture (B).

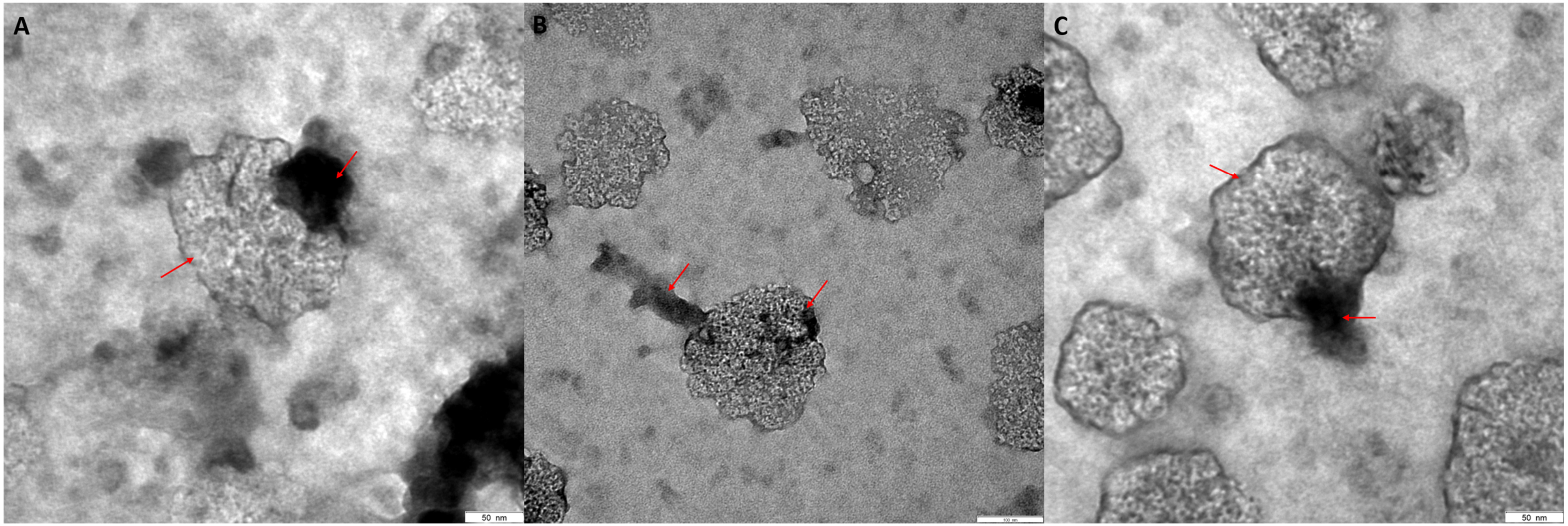

Supplementary Figure S2: Transmission electron micrograph (stained with uranyl acetate, 250 000x magnification) of the bacteriophages. Red arrows indicate the tail (dark) and head of the bacteriophages.

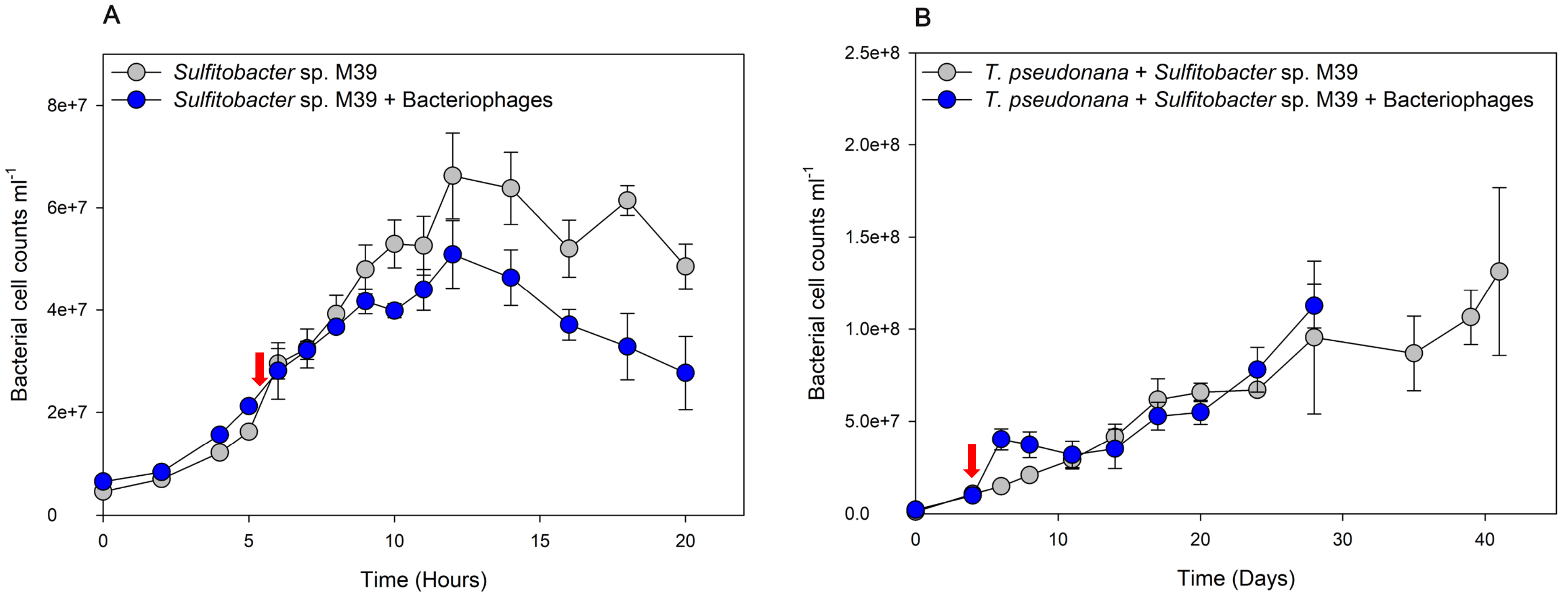

Supplementary Figure S3: ***Sulfitobacter*** sp. M39 cell counts without and with addition of bacteriophages, in absence (A) and presence of ***Thalassiosira pseudonana*** (B). Error bars indicate the standard deviation of triplicates and red arrows indicate the time of bacteriophage lysate addition.
